# Demographics factors and food consumption of diabetic patients type 1 in outpatient care of a Federal University Hospital

**DOI:** 10.1186/1758-5996-7-S1-A238

**Published:** 2015-11-11

**Authors:** Valzimeire do Nascimento de Oliveira, Abelardo Barbosa Lima Neto, Ana Priscilla Silva de Souza, Thaís Cavalcante Façanha, Renan Magalhães Montenegro, Maria Izabel Florindo Guedes

**Affiliations:** 1 Universidade Estadual do Ceará, Brazil

## Background

Diabetes mellitus (DM) has been cited as the most challenging health problem in the 21st century. Successful management of this disease requires that we understand the lifestyle, attitudes, family and social networks of the patients being treated.

## Objectives

To assess the sociodemographic, dietary practices and current food consumption among males and females with type 1 diabetes mellitus.

## Materials and methods

A case-control, descriptive study was conducted in the city of Fortaleza, Brazil, applying a survey on sociodemographic, lifestyle, feeding behavior and food consumption in people treated as diabetic outpatients at the Federal University Hospital. The food consumption was collected through recall 24 h.

## Results

15 individuals were interviewed (53% male; 47% female). Mean of age was 31.3 yrs. Among educational levels was observed that 40% had finished high school and 27% had uncompleted primary education. Analyzing the occupation 40% are students or housewives and 33% are retired or self-employed. Evaluating the lifestyle, 80% do not smoke and drink alcohol. However, 73% of individuals are sedentary. Dietary aspects revealed that 73% follow a food plan, which were oriented: 73% by Nutritionist, 20% for Medical and 7% follow their own guidelines. In addition, 33% performs specific diet for diabetes prescribed by a nutritionist, another 20% follow carbohydrate counting diet, 13% just do not eat sugars and food sugars and 27% do not follow a specific diet for diabetes. Most of patients, 73% answered “respect the amount of food in the diet” when questioned about the difficulties of adhering to diet.” The food consumption of the group was characterized by a deficit in energy intake, adequate carbohydrate percentage energy contribution, excessive in protein, cholesterol and saturated fat. While, polyunsaturated and monounsaturated fat had presented a very low consumption. Fibers consumption proved to be suitable, however it was found deficiency of vitamin D, E and folato.

## Conclusion

Sociodemographic studies like this are fundamental to improve the understanding of lifestyles and dietary practices of individuals with diabetes mellitus 1, being useful in proposing a better treatment strategy for these patients. Preliminary results in general show that the group requires actions of nutritional intervention to correct the dietary inadequacies, because the eating pattern indicates risk to health.

